# Supplementary material for: Challenges and Innovations Brought about by the COVID-19 Pandemic Regarding Medical and Pharmacy Education Especially in Africa and Implications for the Future
Source: Healthcare (Basel). 2021 Dec 13;9(12):1722. doi: 10.3390/healthcare9121722 (PMC8701006; doi:10.3390/healthcare9121722)
Supplement: Supplementary file 1 [file healthcare-09-01722-s001.zip › healthcare-1432219-supplementary.pdf]

Supplementary Tables

Table S1 – Summary of activities and challenges among faculty and students across Africa in response to the COVID-19 pandemic

Location	Experiences and Challenges
**North Africa**	• In Egypt:<br>o Among the medical schools, 84.6% of participants in a survey reported their schools in Egypt were using alternative teaching methods in response to the pandemic [1]<br>o Shehata *et al.* (2020) reported trials of using virtual platforms for teaching and practicing physical examination skills and procedures among medical students in Egypt to improve the experiences [1]<br>o Zalat *et al.* (2021) reported that the greatest barriers to e-learning included insufficient/ unstable internet connectivity (40%), lack of ICT/ support and technical problems (32%). However, there was perceived usefulness, ease of use and acceptance of e-learning among medical students [2]<br>o The Supreme Council for Universities in Egypt has developed a framework for assessing medical students during the pandemic as there is increasing anxiety across the region regarding future employment with the pandemic possibly negatively impacting on competences [1,3,4], with similar situations among other African countries [5]<br>• In Libya, there have been challenges with e-learning, with one third of medical students reporting they had weak internet connections and a quarter did not own ICT devices at the start of the pandemic [4]<br>• In Sudan:<br>o There have been concerns with Pharmacy education as a consequence of the pandemic with only 29.2% of surveyed students having good communication with their colleagues during the pandemic [6]<br>o Concerns with E-learning among medical students in Sudan included unfamiliarity with E-learning systems certainly initially, limitations with internet bandwidth and connectivity, issues with technical support, lack of face-to-face interactions and concerns with time flexibility in case of technical problems during online exams [7]
**East Africa**	• There have been considerable concerns with the cost of the Internet to undertake distance learning classes, concerns with the strength of mobile networks as well as concerns with gaining clinical experience during the pandemic requiring innovative approaches with constant monitoring of new approaches among East African countries [8–11]<br>• In Uganda:<br>o Olum *et al.* (2020) found that 39% of medical students lacked ICT devices, with a similar percentage only having moderate quality internet and up to one-third having low quality internet facilities [12]<br>o Internet costs were also a potential barrier to e-learning among 93% of HCP students surveyed at Makerere University with reliable electricity also a barrier to learning (56%) [12]<br>• Despite these challenges, generally among East African countries e-learning has had a positive impact on the learning of clinical skills among students [12,13]<br>• In Rwanda in some hospitals, robots have taken over patient monitoring and documentation of care during the pandemic alongside the increasing the use of drones and telemedicine to improve patient care [14]. These experiences provide direction for the future skills of HCPs across Africa

**Central Africa**	• There have also been challenges and activities among Central African countries in response to the COVID-19 pandemic<br>• This included the University of Yaoundé in Cameroon with early challenges to centralise its courses to an online platform [15]<br>• In the Democratic Republic of Congo, clinical rotations for Medical and Pharmacy students were suspended following the first registered COVID-19 case [16]. However, other forms of education have now taken over including a rise in e-learning [17,18]
**Southern Africa**	• In Botswana, the pandemic gave an opportunity for the Department of Family Medicine and Public Health Medicine at the University of Botswana to instigate greater service-learning [19]. This includes greater opportunity for health science students to connect services with the academic curriculum to improve future skills<br>• In Eswatini - The costs of internet access, ICT infrastructure, reliable electricity and access to computers have been a challenge among tertiary students, which is now being addressed [20]<br>• In Malawi, the institutions of higher learning were slow in migrating from face-to-face teaching to online teaching exacerbated by interrupted electricity, internet connectivity issues, funding issues for the internet and associated bundles as well as insufficient ICT equipment to aid on-line teaching when the pandemic first hit Africa [21,22]. However, this is changing<br>• In Namibia, considerable efforts have been made to optimise Pharmacy education during the pandemic. Pro-active interventions include improving competencies regarding online education and training and identifying ten key elements for the successful implementation of a resilient pharmacy education programme during the pandemic [23,24]. This was in addition to ensuring continued pharmacy graduate compounding skills during the pandemic as well as assessing pharmacists' readiness to adequately respond to the pandemic [25,26]<br>• In South Africa:<br>o There have also been concerns with reliable electricity supply, limited network coverage, Internet access, availability of data and other challenges among students and teaching staff, affecting emergency remote teaching (ERT)/e-learning [27,28]. In addition, access to online teaching and learning platforms as well as resources for students from poorer rural communities across South Africa [29]<br>o Lockdown measures have also prohibited students in South Africa from completing compulsory practical and experiential training as required by their professional bodies for completion of their qualifications, posing challenges to universities to retain accreditation with these professional bodies [28]. To address this, instructors in South Africa have created a predictable course structure and reach out to struggling students to help with their education [30]<br>o These challenges in South Africa have resulted in some universities sourcing ICT devices for their students and staff as well as purchasing these at preferential rates, with other universities obtaining external sponsorships to cover such costs [28,31]<br>o Universities in South Africa have also instigated additional security measures to enable students to take their examinations remotely whilst ensuring the quality and integrity of assessments [31]<br>o It is also increasingly accepted in South Africa that transitioning e-learning and assessments online demanded creativity and patience to achieve the necessary objectives. A key benefit though arising from these circumstances in South Africa and across Africa has been highlighting shortcomings in the various healthcare

	systems and pointing out ways that pharmacists and other HCPs can improve care in the future thereby further improving patients' lives [30]<br>• In Zimbabwe, there have also been considerable concerns with e-learning and conducting examinations online due to expensive internet access, poor connectivity and electricity shortages [32]
**West Africa**	• Among Western African countries including Ghana and Nigeria, there have also been closures of Medical and Pharmacy schools at the onset of the pandemic [33]<br>• This was a concern initially among Pharmacy schools in Nigeria as pharmacy educators and schools were under-equipped initially for any e-learning [34]. In addition, students' inability to regularly access the Internet results in concerns with their education [34,35]<br>• There have been similar issues among medical students in Nigeria [5,36]; however, this is starting to change

NB: ICT = Information and communications technology; HCP = Healthcare Professionals

Table S2 – Challenges faced by Universities and students across Africa as a result of the pandemic

Country	Challenges
**Botswana**	***a) Teaching and learning challenges included:***<br>• Interruption of the academic calendar because of national lockdowns and students travelling to their homes – a concern as a number of students did not have access to learning materials, e.g., suggested books<br>• Little experience initially with e-learning and teaching among both students and faculty members<br>• Whilst the majority of the students did possess a smartphone, few had ICT devices at their homes to support e-learning. Learning or teaching using smartphones consequently became the principal option – although causing challenges certainly initially<br>• Not all students had internet facilities to support leaning online, and there were concerns with its reliability<br>• Whilst mobile data provided by the University of Botswana was the source of internet connectivity for most students, this was often not strong enough to sustain online learning/teaching<br>• Rapid instigation of effective problem-based learning (PBL) through initiating online platforms to address the lack of face-to-face learning opportunities<br>***b) Resource and other challenges included:***<br>• The cost of the internet was also prohibitive for most students and some faculty members accentuating the challenges<br>• Poor internet connectivity challenging the running of online tests
**Cameroon**	***a) Teaching and learning challenges included:***<br>• Lectures had to be adapted to online learning which resulted in challenges with limited prior experiences among staff and students [15]<br>• Some lectures that continued face-to-face also had challenges of social distancing as well as water sanitation and hygiene issues. Access to water is a major challenge in many learning institutions in Cameroon, and the cost of masks and hand sanitizers prohibited poorer students from attending classes<br>***b) Resource and other challenges included***:<br>• Online learning itself was challenging due to the costs of internet data bundles, lack of smart phones among poorer students, and the reliability of internet connections<br>• Some students who interrupted their classes due to COVID-19 did not return after lockdown measures were eased
**Eswatini**	***a) Teaching and Learning challenges included:***<br>• Interruption of the academic calendar which eventually led to delayed progression and graduation of final year students.<br>• The conventional in-person contact lectures, group & individual presentations could not take place as a result of the pandemic. This led to the introduction of remote or online learning<br>• However online learning evolved with its own shortcomings including challenges with gadgets (e.g., laptops, smartphones), literacy on the use of different learning platforms, internet connectivity quality, data cost and power outages<br>• Visitation by guest lecturers and industrial tours for students were also cancelled<br>• Laboratory practicals and hands-on learning was challenged, consumables for laboratory practicals could not be sourced due to closure of shops.<br>• Clinical rotation/internship exposure at various clinical sites for students was suspended (students and supervisors)<br>• Community services such as public enlightenement campaign on pharmacy education could not be fully rendered

	• The quality of assessment (e.g., tests and semester examinations) was compromised. Continuous assessments replaced final semester examination for junior levels.<br>***b) Other challenges included:***<br>• Final year students had limited access to health facilities for data collection while primary research designs were substituted with secondary desktop reviews
**Ghana**	***a) Teaching and Learning challenges included:***<br>• Lockdown measures impacted on the mode of delivery of lectures with practice-based courses modified into virtual learning courses to reduce student-student and student-lecture contact in health science universities<br>• Both prior to and after the initial lockdown, universities had limited capacity to adequately test students before their departure home and upon their arrival to continue studies and take examinations. This greatly impaired university efforts to ensure students were adequately monitored with special provisions made where concerns. This coupled with a lack of isolation or quarantine facilities meant students did interact in places of accommodation where protective and preventive measures could not be properly enforced, which was a concern<br>• Practical assessments for laboratory-based courses and hospital-based courses had to be converted into objective structured clinical examination (OSCE) format - affecting clinical skill-based learning<br>• Large class sizes (some usually in excess of 50 students) presented difficulties regarding online learning and social distancing measures when students were back attending lectures in person<br>• Some examinations were postponed indefinitely until students were able to report back to universities to take them<br>• The performances of a number of students have been reduced with many distractors when learning from their homes<br>***b) Resource and other challenges included***:<br>• Smooth delivery of online lectures has been a challenge with some students unable to afford data bundles to participate in all lectures<br>• Poor internet access and networks in students' home affected the smooth delivery of uninterrupted lectures<br>• There were instances where some students acquired COVID-19 affecting their health status and ability to undertake e-learning courses
**Kenya**	***a) Teaching and Learning challenges included:***<br>1. Interruption of the academic calendar due to extended lock down and closure of universities in the wake of the COVID-19 pandemic – this eventually led to delayed academic progression of students and disruption of student placements and experiential rotations<br>2. Initial resistance by staff and students to online teaching and e-learning; most questioned the effectiveness of this approach initially. This was exacerbated by:<br>3. anxiety, apprehension and confusion among the learning institutions and government certainly initially due to a lack of policies in managing teaching and learning during global pandemics<br>4. lack of access to expertise and guidance from senior experienced clinical staff since those above 55 years of age and considered highly vulnerable to COVID-19 infection were put on strict instructions not to have any physical interaction with students<br>5. Delays in the completion of the curricula as the university was closed at the start of the pandemic exacerbated by delayed resumption of teaching and learning whilst awaiting the development, approval and release of COVID-19 containment guidelines by the Ministry of Health and guidelines on virtual teaching and learning by the University<br>6. Reduction in the number of physical classes and reduced personal interaction with students enhanced by the lack of adequate physical space and resources to handle small groups of students and ensure efficient and timely delivery of teaching and learning while adhering to

	COVID-19 containment measures. This was exacerbated by concerns with exposure of staff and students to the virus, some of whom were infected and had died from COVID-19 (not in pharmacy but in the college of health sciences); this increased anxiety levels<br>7. Challenges in conducting laboratory experiments while enhancing social distancing for large groups of students. This included an inability to effectively conduct bedside/ward/clinical teaching given the large numbers of students and fear of contracting the disease<br>8. Delays or gaps in conducting clinical research with specific patient groups in the wake of the pandemic, sometimes occasioned by lack of suitable patients with many avoiding hospitals in the wake of the pandemic<br>9. Concerns with performing examinations for large groups of students and assurance of online examination integrity<br>***b) Resource and other challenges included***:<br>***1.*** Lack of access to the internet, its reliability and computers among students, especially those from low-income settings
**Malawi**	***a) Teaching and Learning challenges included:***<br>2. Disruption of the academic calendar with subsequent impact on clinical placements and dissertations for the students not helped by fear by many staff members who opted to work from home led to delays in decision making<br>3. Shortage of classrooms (maximum number of 50 per gathering and sitting at least 1 m apart) leading to splitting of classes when over the limit, increasing the workload for teaching staff<br>4. Shortage of staff and absenteeism from work due to morbidity/ mortality among staff members and their relations/ neighbours further exacerbating the situation<br>5. Panic, uncertainties and communication lapse in institutions due to frequent changes in restrictions by the government as the changes were dependent on many factors locally and internationally<br>6. Cancelling or delaying of some institutional activities that are mostly undertaken outside the institutions such as curriculum development and reviews and many other community engagement projects by the institution. This was exacerbated by some practical attachment areas have refused to allow Pharmacy and Medical students for attachments due to COVID-19 - except government and Christian Health Association of Malawi (CHAM) facilities - negatively impacting on students as they will lack valuable practical knowledge and experience at graduation<br>7. Shortage of laboratory space in order to accommodate social distancing rules – resulting in the splitting of classes, leading to more days for practicals and fewer lab practicals overall negatively impacting on students' learning<br>***b) Resource and other challenges included***:<br>1. Limited funds to implement some of the measures and restrictions set by the government as well as poor internet access and connectivity for staff members and students<br>2. Challenges in access to gadgets such as computers and tablets for the students<br>***3.*** Wastage of reagents with short stability because of prolonged number of days to complete a practical and shifting of laboratory sessions to the following week(s). In addition, delays in acquiring solvents and reagents for students and research activities as a result of the pandemic
**Namibia**	***a) Teaching and Learning challenges included:***<br>1. There have been numerous challenges since Health Sciences education, especially medicine and surgery, are based on the principles and practice of ''apprenticeship'' which involves physical contact between teachers and students for the acquisition of clinical skills in particular. Alongside this, the teaching of anatomy relies heavily on cadaver dissection and use of anatomical specimens, which was not possible during the COVID-19 pandemic [37]<br>2. However, there has been a growth in computer-based models and other approaches to address concerns given the recognised importance of for instance dissection classes [38,39]

	3. A number of learning activities were put on hold following the outbreak of COVID-19 pandemic; however, the extent of knowledge gaps varied by institution depending on their infrastructures and knowledge of e-learning before the pandemic. Many private educational institutions were able to resume some form of e-learning quickly as opposed to government-owned institutions, which typically started much later due to the greater availability of e-learning platforms among privately-owned institutions before the current pandemic [40]<br>4. There has been re-scheduling of classes among institutions especially government-owned institutions as a result of the pandemic, with teaching staff aware of distractions in home environments<br>5. Students typically embark on ''clerkships'' involving interactions with patients which became a challenge during the pandemic. Telemedicine approaches may potentially help, with the use of telemedicine increasing during the pandemic to avoid increasing transmission rates [41–43]<br>6. The mandatory laboratory based practical sessions for pre-clinical studies were also put on hold initially, although this is now changing<br>7. The academic sessions became prolonged as a result of the general closures of educational institutions and the need to make up for missed clinical postings and laboratory sessions, which are compulsory components of the curricular<br>***b) Resource and other challenges included***:<br>• Poor internet connectivity, unaffordability of the cost of data bundles among students, and inadequate interactions with teachers
**Nigeria**	***a) Teaching and Learning challenges included:***<br>• There have been numerous challenges since Health Sciences education, especially medicine and surgery, are based on the principles and practice of ''apprenticeship'' which involves physical contact between teachers and students for the acquisition of clinical skills in particular. Alongside this, the teaching of anatomy relies heavily on cadaver dissection and use of anatomical specimens, which was not possible during the COVID-19 pandemic [37]. However, there has been a growth in computer-based models and other approaches to address concerns given the recognised importance of for instance dissection classes [38,39]<br>• A number of learning activities were put on hold following the outbreak of COVID-19 pandemic; however, the extent of knowledge gaps varied by institution depending on their infrastructures and knowledge of e-learning before the pandemic. Many private educational institutions were able to resume some form of e-learning quickly as opposed to government-owned institutions, which typically started much later due to the greater availability of e-learning platforms among privately-owned institutions before the current pandemic [40]<br>• There has been re-scheduling of classes among institutions especially government-owned institutions as a result of the pandemic, with teaching staff aware of distractions in home environments<br>• Students typically embark on ''clerkships'' involving interactions with patients which became a challenge during the pandemic. Telemedicine approaches may potentially help, with the use of telemedicine increasing during the pandemic to avoid increasing transmission rates [41–43]<br>• The mandatory laboratory based practical sessions for pre-clinical studies were also put on hold initially, although this is now changing<br>• The academic sessions became prolonged as a result of the general closures of educational institutions and the need to make up for missed clinical postings and laboratory sessions, which are compulsory components of the curricular<br>***b) Resource and other challenges included***:<br>• Poor internet connectivity, unaffordability of the cost of data bundles among students, and inadequate interactions with teachers
**South Africa**	***a) Teaching and Learning challenges included:***

	• Maintaining pedagogical quality and rigor for students with diverse learning needs and ethnically diverse student populations, ensuring effective teaching and student engagement, developing reliable and valid assessment methods for practical skills/ clinical assessments, maintaining the quality and integrity of online assessments, and continuing experiential education without compromising student or staff safety or learning outcomes<br>• Diversity in terms of race, cultural identities, languages, socioeconomic backgrounds (especially rural areas) and those with disabilities, such as visual impairment, among some of the Medical and Pharmacy schools in South Africa have presented challenges in accessing and adopting online platforms, which is now being addressed<br>• Further challenges come from the shift within the environmental context from which education has moved from the relative stability, and perhaps sterility, of campus life to the very real context of entering, witnessing, and at times experiencing student's home life, with students also forced into different roles of responsibility in the home environment. This also needs to be taken into context with any e-learning approach<br>• Shortened academic year as a result of lockdown measures, a risk-adjusted strategy with phased-in return of students and the time required to develop new or hybrid teaching and learning strategies<br>• Training of academic staff towards e-learning and platforms where teaching was predominantly traditional had to be accelerated in addition to the instigation of the necessary infrastructure to facilitate online assessments to minimise disruption from the pandemic<br>• Infrastructure of academic institutions and ensuring compliance with occupational health and safety requirements to allow the phased-in return of staff and students e.g., screening, personal protective equipment (PPE), sanitation, monitoring and isolation facilities<br>***b) Resource and other challenges included***:<br>• Financial constraints and inequalities among higher education institutions, and within institutions among students, hampered smooth and prompt transition from face-to-face to online teaching and learning, which came unexpectedly as a result of the pandemic. This has been exacerbated by already severely constrained academic human capacity for several years prior to COVID-19 among many academic institutions<br>• Concerns with access to the internet [27,28], as well as access to online teaching and learning platforms and general resources for students from poorer rural communities across South Africa [29]<br>• Negative implications of the pandemic on the performance, mental health and well-being of students and academic staff, which hampered progress on transforming models of teaching and learning<br>• Personal stressors included extended working hours for staff with no breaks or weekends away, imbalances between research and teaching responsibilities, inexperience in the online transition, feelings of isolation, childminding, home-schooling for children, household management, vulnerable students' living conditions which did not allow studying at home and meaningful participation in remote teaching and learning, e.g., small living spaces occupied by extended family members
**Tanzania**	***Teaching and Learning challenges included:***<br>• Universities were still teaching face-to-face in the early stages of the pandemic. However, all students must wear a face mask which is mandatory also for lecturers<br>• All lecture theatres and laboratories, even clinical attachment places, were supplied with sanitizers and running water with soap - now mandatory. Staff or students who failed to comply with these regulations were denied entry to campuses<br>• University wide meetings including senate meetings were no longer held face-to-face – but via the Internet with associated challenges
**Uganda**	***a) Teaching and Learning challenges included:***

	• General resistance by staff and students to embrace online teaching and e-learning as a viable alternative avenue for teaching and training in the country<br>• Effective administration of examinations for different groups of students while ensuring examination integrity and fairness to students.<br>• Whilst the government allowed some university education in Medical Schools, there have been serious challenges and difficulties with effective delivery of clinical and bedside teaching and instructions in the clinical teaching areas<br>• A real fear that students will be exposed to COVID-19 and risk infection during their training with a real possibility of Universities and hospitals being overwhelmed by infected personnel<br>***b) Resource and other challenges included***:<br>• Challenges with providing e-learning facilities at home due to poverty, very poor mobile network penetration, high cost of internet data for online access, poor knowledge on how to use/operate online platforms, and unreliable electricity supply to power devices for online platform access [12]<br>• Lack of online teaching infrastructures certainly initially, including internet access, computers, and mobile devices as well as lack of access to reliable electricity or solar energy supply among training institutions
**Zambia**	***a) Teaching and Learning challenges included:***<br>• Restrictions on face-to-face, contact-based learning and challenges of implementing e-learning:<br>o In Zambia, pharmacy and medical education programmes have largely utilised traditional (contact-based, face-to-face) approaches to facilitating learning in universities, including experiential learning undertaken in tertiary teaching hospitals [44]. COVID -19 has impacted on local capacity to deliver traditional teaching<br>o In March 2020, all universities were instructed to resort to e-learning using online platforms. However, there have been challenges with the e-learning infrastructure. In addition, understanding what constitutes successful e-learning is an important first step among training institutions having to deal with their effectiveness<br>o Whilst there have been improvements in the availability of the internet, access to, and quality of, the internet remains a challenge<br>• Barriers to access e-learning by students:<br>o Inadequate capacity to deliver online teaching certainly initially<br>o Resistance to change by both faculty and students to the utilisation of online facilities for teaching<br>o Adherence to policy regulators requirement for changes to approved teaching methods<br>• Limited clinical and practical skills teaching:<br>o Skills labs in Zambian universities were inadequate at the start of the pandemic with clinical teaching largely relying on using teaching hospital settings. This is a concern with public hospitals imposing restrictions on the number of students that can be in the hospital wards at any given time. In addition, several tertiary teaching hospital general wards had been converted to COVID-19 wards with restricted access to students. Consequently, practical teaching of clinical level medical and pharmacy students has been significantly affected<br>o A recent survey of nursing science students in Zambia showed that due to challenges posed by COVID-19 pandemic on their education, the majority were unable to complete their course work resulting in some concepts being poorly grasped and they faced delayed final examinations. In addition, internet facilities were often poor resulting in missing class lessons and assessments [45]<br>• Challenges to conducting assessments:

	o Assessing clinical skills using such methods as Objectively Structured Clinical Exams (OSCE) is challenging amidst COVID-19<br>o A concern is that assessing clinical skills via e-learning platforms remains potentially unreliable and technologically challenging, which needs to be addressed<br>***b) Resource and other challenges included***:<br>• Psychosocial and mental health wellbeing of students:<br>o Needs to be continually assessed and addressed given the self-motivation needed to optimise e-learning<br>o This is because the COVID-19 pandemic has impacted negatively on the mental health of pharmacy students in Zambia with a recent survey suggesting 75% of students experienced anxiety about COVID-19. Factors that affected mental health included; reduced family care, reduced time of resting, and feeling helpless [46]<br>• Barriers to access e-learning by students:<br>o Inadequate internet capacity and accessibility by students and faculty<br>o Despite students not expressing misgivings about e-learning, barriers to accessing it can be a challenge that is beyond institutional control, but situated in the wider context in higher education provision in Zambia<br>• The many issues need to be addressed going forward with e-learning in Universities here to stay [47]
**Zimbabwe**	***a) Teaching and Learning challenges included:***<br>• Routinely establishing learning platforms for both staff and students necessitating education among both<br>• Adapting to different approaches to practical sessions and clinical teaching with for instance patients typically discharged from learning hospital institutions in preparation for COVID-19 cases reducing clinical learning experiences<br>***b) Resource and other challenges included***:<br>• Students having to acquire smart electronic gadgets for e-learning at their own expense – causing considerable variation in their abilities to undertake e-learning<br>• Internet connectivity was not homogenous among learning institutions and students<br>• Inadequate attention to the psycho-social wellbeing of students

NB: ICT = Information and communications technology

Table S3 – Summary of responses among institutions across Africa to the pandemic and its impact on educational approaches for physicians and pharmacists

Country	Response
Botswana	***a) Responses to teaching included:***<br>• The extensive use of electronic remote teaching, with virtual learning continuing after the opening of the university<br>• Accessible soft copies of reference textbooks were sent to some students in their home where finances were a problem<br>• Regular adjustments of teaching schedules to accommodate students on quarantine due to COVID-19<br>***b) Responses to practicals and clinical teaching included:***<br>• The approach to some elective modules changed to limit contact with patients or groups of other people, e.g., the routine year 4 elective (Medicine) that often post students to clinical departments for attachments was modified with students provided with PPE for this as well as for resuming their clinical duties. This was in contrast to the pre-COVID-19 situations where electives were performed with students attached to specific departments and discussions conducted face-to-face<br>• Students conducted systematic reviews in small groups as part of fourth year activities (Medical students) with assistance of faculty members. Most activities were virtual in contrast to pre-COVID-19 activities
Cameroon	***Responses to teaching included:***<br>• Use of WhatsApp® forums to disseminate lectures was the sole approach available certainly initially for distance learning. The asynchronous approach was preferred by many students due to lower cost of using data on WhatsApp® and the relative ease with using WhatsApp®<br>• Some lecturers continued with face-to-face classes with social distancing measures instigated to help keep students and staff safe. This included compulsory use of face masks for entry onto university campuses with compulsory testing for COVID-19 instituted later during the pandemic (early 2021)
Eswatini	***a) Responses to teaching included:***<br>• Remote or online learning introduced to overcome stopping of face-to-face lectures. A blended learning approach was subsequently adopted following several senate deliberations after the easing of lockdown restrictions<br>• Academic staff were trained on the use of google classroom as the official online learning platform and user manuals were made available for both students and lecturers. Other online learning platforms including WhatsApp® groups, Zoom®, Teams and Google Meet were also utilised by some instructors based on convenience with the students<br>• The university made provision for hand washing and sanitizing stations at various areas on the campus. In addition, it became compulsory for students and staff to wear a face mask before entry onto the university and for any practical laboratory sessions<br>• Temperature checks were also undertaken at the campus entrance and in laboratories as well to enhance safety<br>***b) Responses to practicals and clinical teaching included:***<br>• YouTube videos and self-made interactive videos were initially use as a substitute to hands-on practical session during the lockdown<br>• Following the ease of lockdown restrictions, the practical sessions arranged in the laboratories were staggered with fewer students and for shorter durations in line with University regulations
Ghana	***a) Responses to teaching included:***

	• The development of an electronic learning management system by universities for uploading of lectures and assignments for students<br>• Some lectures were also pre-recorded for students who could not be present during online classes to download and watch at later times<br>• Implementation of COVID-19 safety protocols by installing hand washing stations and hand sanitizers at various points in the university when students returned and enforcement of mask-wearing during face-to-face lectures. This was helped by some Pharmacy schools scaling up their production of hand sanitizers and face masks to meet the demand from universities (and also the public)<br>***b) Responses to practicals and clinical teaching included:***<br>• Practical assessment of students for laboratory-based courses and hospital-based courses were converted into an objective structured clinical examination (OSCE) format following the suspension of all onsite clinical training courses
Kenya	***a) Responses to teaching included:***<br>• Early instigation of webinars and presentations on COVID-19 to create awareness, provide education, promote vaccine uptake and other preventative measures among students and staff<br>• Establishing early the College of Health Sciences and School of Pharmacy COVID-19 committees to undertake these and other activities as well as provide free vaccinations for students and staff<br>• The transition from physical lectures to online lectures with minimal face-to-face lectures was assisted by investment in, and subscription, to online teaching platforms. Alongside this, extensive training of students and staff on various models of online teaching and examination including Google meet and Zoom®<br>• Revision of teaching timetables where there were delays to fast track teaching and completion of the curricula<br>• Provision of internet bundles to students and staff by the University of Nairobi to enhance digital teaching<br>***b) Responses to practicals and clinical teaching included:***<br>• Introduction of innovative methods of clinical teaching including using videos and dividing students into small groups for clinical and laboratory teaching<br>• Training of students and staff on the software and examination process to ensure examinations are undertaken effectively and integrity maintained<br>• Provision of PPE to students and staff conducting clinical/bedside teaching and learning; provision of additional handwashing stations with soap and water<br>• Provision of adequate accommodation for students taking practical courses during the pandemic
Malawi	***a) Responses to teaching included:***<br>• Introduction on online learning with the aim of using an institution specific adapted online system, the COMPASS<br>• Training of staff and students on the use of online teaching and learning platforms<br>• Working from home policy for all members of staff and the introduction of shift working system for staff that cannot work at home entirely<br>• Staggered or incremental opening of the colleges, blending of face-to-face and online learning and splitting of classes into various streams to comply with current regulations as lockdown measures were eased<br>• Issuing of internet bundles to the staff to assist with working from home. However, this measure led to other challenges such as the appreciable extra cost on the university institutions to purchase internet bundles for staff and students, which was later found to be unsustainable and stopped

	• Alongside this, active discussions with telephone network operators to provide cheaper internet access for students
Namibia	***a) Responses to teaching included:***<br>• The University of Namibia developed a policy and strategic plan aligned to the Government of Namibia's COVID-19 protocols to sustain education during the pandemic<br>• The migration of all learning to remote, online learning. The primary online platform for teaching and learning was Moodle, though lecturers used various platforms including Zoom®, MS Teams, Google Meet and WhatsApp® to advance student-lecturer interaction<br>• Within two weeks of moving to teaching online, a survey of all Pharmacy students was conducted to identify challenges to access online learning materials, which had a response rate of 95% and showed that all students could access Moodle but 29% of students could not access Moodle all the time [48]. This information was important to guide the lecturers' decision making with regards to timing of assessments and requirements for synchronous teaching.<br>• One staff member with experience in using Moodle was appointed as the de-facto coordinator for online teaching and learning for the School of Pharmacy. In the first semester following the start of the pandemic, all theoretical examinations were replaced by online formative assessments completed via the Moodle platform, with Moodle helping to smooth the process from face-to-face to fully online teaching.<br>***b) Responses to practicals and clinical teaching included:***<br>• The practical and clinical training in some of the health sciences including Pharmacy were shifted to online recorded sessions or patient case study discussions using a problem-based learning approach<br>• The university calendar was amended to allow for remedial as well as practical and assessment intervention periods to ensure all students had the opportunity to complete their modules and progress to the next academic year<br>• Practical training was conducted in smaller batches of students in order to comply to COVID-19 regulations<br>• Final year Pharmacy and Medical students were allowed to return to University hostels in order to complete clinical rotations under safe conditions. Alongside this, clinical research projects for final year students were transformed to systematic reviews where possible and more recently, clinical presentation assessments have been conducted online combined with the use of workbooks<br>• The industrial pharmacy experiential learning module was modified to an in-house training with help of local institutions such the Ministry of Health and Social Services Quality Surveillance Laboratory and National Regulatory Authority<br>• Experiential learning modules in Rural, Community and Hospital pharmacy were completed by all students by virtue of being flexible with regards to timing, duration and mechanisms used for the modules
Nigeria	***a) Responses to teaching included:***<br>• Most privately-owned institutions quickly commenced online classes - including lectures, seminars conducted as webinars, thesis defense and examinations following closure of Universities. Government-owned facilities were slower in starting online classes but most eventually commenced e-learning approaches using various platforms including Zoom®, Google Classroom or Microsoft Teams<br>• As soon as phased re-opening of the lockdown commenced, Medical and Pharmacy schools resumed and the following activities were also introduced:<br>• Hybrid learning sessions in some institutions with students having the option of resuming physical classes or remaining at home to continue online<br>• Training in the use of e-learning platforms organised early for academic staff in many universities to enhance the quality of teaching and learning

	• Courses with large number of students were broken down into smaller groups and taught at different times or simultaneously using networked projectors to multiple lecture rooms<br>• Public awareness of COVID-19 and its prevention - displayed as posters at entrances to academic and other buildings within educational institutions<br>• Enforcement of hygienic practices and compulsory use of face masks within educational institutions<br>• All educational institutions had a committee to ensure compliance to COVID-19 protocols with supervision from the Ministry of Education<br>***b) Responses to practicals and clinical teaching included:***<br>• For clinical students, attendance at clinics and ward rounds were broken down into batches to ensure physical distancing and compliance with other prescribed preventive measures<br>• Re-scheduling of practical sessions in batches of fewer numbers of students per sub-group to conform with COVID-19 measures including social distancing and optimization of the relatively limited laboratory equipment and infrastructure
South Africa	***a) Responses to teaching included:***<br>• The most popular approach involved shifting curricula onto online platforms. This included teaching, assessment, as well as practicals conducted by showing student the techniques via video and simulations – although recognising significant challenges in technological readiness and the availability of critical resources to undertake this approach in a way that did not marginalise students<br>• In addition, guidelines were established for how educational resources should be compiled and the infrastructure upgraded to accommodate a greater need for digital resources<br>• Zero-rated data organised with telecommunications to assist with supporting online teaching and learning, especially among students, as well as acquisition of laptops for distribution to students<br>• Strategies by universities to ensure learning by facilitating the return of vulnerable students and those living in remote areas as a priority when a limited number of students were allowed to return to campus. Along with this, clinical training platforms were staggered to accommodate safety protocols or shifted to minimum requirements wherever possible<br>***b) Responses to practicals and clinical teaching included:***<br>• Shortening the time period and reducing the size of student groups where there is work-based learning in primary healthcare facilities, community pharmacies and hospitals were allowed<br>• The regulatory body for pharmacists, the South African Pharmacy Council (SAPC), assisted the change to online platforms by amending the 400 hours of compulsory work-based learning to include work-integrated learning<br>• Quality assurance of the pharmacy qualification by SAPC in collaboration with Pharmacy Schools to ensure that final-year students were able to complete their course in time to commence with internships in January 2021 in order to prevent any negative impact on the healthcare workforce as a result of the pandemic<br>• For clinical training - acquisition of PPE for students and staff members
Tanzania	***a) Responses to teaching included:***<br>• Teaching continued to be face-to-face; however, students and lecturers had to wear face masks<br>• University meetings including senate, council and staff meetings to discuss teaching and related subjects were held virtually via ZOOM®<br>***b) Responses to practicals and clinical teaching included:***

	• Conducting tests and examinations were still held face-to-face; however, everyone had to wear a face mask and regularly clean their hands with running water with soap is supplied at all teaching facilities<br>• For clinical attachments, laboratories, wards and theatres everyone must wear a face mask and regularly clean their hands with soap and running water
Zambia	***a) Responses to teaching included:***<br>• Universities and Colleges offering pharmacy and medical training responded to the COVID-19 pandemic by adopting alternative means of learning through e-learning platforms including Moodle® and Astria®.<br>• In Zambia, blended learning was initially instituted using periodic alternation between learner groups in the universities. Following the rise in COVID-19 cases, the government on June 17, 2021 imposed further lockdowns and instructed all universities to resort to online teaching until the situation improves – as result all higher education institutions are currently expected to provide e-learning<br>• Initiatives such as the Zambia Educational and Research Network (ZAMREN) as well as resources through e-libraries and research collaboration have provided member institutions education roaming (e.g., Eduroam) and cloud computing. This has facilitated connectivity to the internet where the service is deployed for every user registered with the member institution within Zambia [49].<br>• The majority of the teaching in health sciences is now undertaken online using various online platforms. This is despite challenges with costs, quality and issues of access to the internet amidst a harsh economic situation
Zimbabwe	***a) Responses to teaching included:***<br>• Free internet connectivity was established at the University of Zimbabwe College of Health Sciences for students in addition to some teaching hospitals. However, this is not universal with one of the biggest hospitals in Zimbabwe (Sally Mugabe Central hospital) still doesn't have internet connectivity for Medical and Pharmacy students who do rotations there<br>***b) Responses to practicals and clinical teaching included:***<br>• Face-to-face teaching continued for clinical rotations – however mindful of the virus<br>• Students had didactic lessons online continuously for a month from 0800hrs to 1600hrs and subsequently back for clinical rotations for a month to reduce risks of COVID-19 infection and keep educational input manageable

NB: PPE = Personal Protective Equipment

References

1. Shehata M.H; Abouzeid, E.; Wasfy, N.F.; Abdelaziz, A.; Wells, R.L.; Ahmed, S.A. Medical Education Adaptations Post COVID-19: An Egyptian Reflection. *Journal of medical education and curricular development.* **2020**, *7*,2382120520951819.
2. Zalat, M.M,; Hamed, M.S.; Bolbol, S.A. The experiences, challenges, and acceptance of e-learning as a tool for teaching during the COVID-19 pandemic among university medical staff *PloS one.* **2021**, *16*, e0248758.
3. Bouali, H.; Okereke, M.; Adebisi, Y.A.; Lucero-Prisno III, D.E. Impact of COVID-19 on Pharmacy Education. *SciMedicine Journal*. **2020**, *2*, 92–95.
4. Alsoufi, A., Alsuyihili, A.; Msherghi, A.; Elhadi, A.; Atiyah, H.; Ashini, A.; Ashwieb, A.; Ghula, M.; Ben Hasan, H.; Abudabuos, S.; et al. Impact of the COVID-19 pandemic on medical education: Medical students' knowledge, attitudes, and practices regarding electronic learning. *PloS one.* **2020**, *15*,:e0242905.
5. Ossai, E.N. Impact of COVID-19 on medical education and the challenges: how prepared is Nigeria? *Pan. Afr. Med. J.* **2020**, *37*, 45.
6. Kamil, N.A.A.; Mohammed, S.E.E.; Elhadi, Y.A.M.; Musa, M.B.M.; Adebisi, Y.A.A.; Prisno III, D.E-L.The impact of COVID-19 on pharmacy students in Sudan: A cross-sectional survey. *Pharmacy Education.* **2020**, *20*, 276–82.
7. Gismalla, M.D.; Mohamed, M.S.; Ibrahim, O.S.O.; Elhassan, M.M.A.; Mohamed, M.N. Medical students' perception towards E-learning during COVID 19 pandemic in a high burden developing country. *BMC medical education.* **2021**, *21*, 377.

8. Wafula, I.; Ong'era, E.M. Deprived of the Sea: Being a Kenyan Final-year Medical Student During the COVID-19 Outbreak. *Int. J. Med. Students.* **2021**, *9*, 80–1.
9. Haileamlak, A. How Should Medical Education Continue During COVID-19 Pandemic? *Ethiop J. Health. Sci.* **2020**, *30*, 855–856.
10. Dawidziuk, A.; Kawka, M.; Szyszka, B.; Wadunde, I.; Ghimire, A. Global Access to Technology-Enhanced Medical Education During the COVID-19 Pandemic: The Role of Students in Narrowing the Gap. *Global health, science and practice.* **2021**, *9*,10–14.
11. Ahmed, S.A.; Hegazy, N.N.; Malak, A.H.W.; Cliff, K.W.3rd.; Elrafie, N.M.; Hassanien, M.; Al-Hayani, A.A.; El Saadany, S.A.; Ai-Youbi, A.O.; Shehata, M.H. Model for utilizing distance learning post COVID-19 using (PACT)™ a cross sectional qualitative study *BMC medical education.* **2020**, *20*, 400.
12. Olum, R.; Atulinda, L.; Kigozi, E.; Nassozi, D.R.; Mulekwa, A.; Bongomin, F.; Kiguli, S. Medical Education and E-Learning During COVID-19 Pandemic: Awareness, Attitudes, Preferences, and Barriers Among Undergraduate Medicine and Nursing Students at Makerere University, Uganda. *Journal of medical education and curricular development.* **2020**, *7*, 2382120520973212.
13. Gormley, G.J.; Collins, K.; Boohan, M.; Bickle, I.C.; Stevenson, M. Is there a place for e-learning in clinical skills? A survey of undergraduate medical students' experiences and attitudes. *Medical teacher.* **2009**, *31*, e6–12.
14. Karim, N.; Jing, L.; Lee, J.A.; Kharel, R.; Lubetkin, D.; Clancy, C.M.; Uwamahoro, D.; Nahayo, E.; Biramahire, J.; Aluisio, A.R.; et al. Lessons Learned from Rwanda: Innovative Strategies for Prevention and Containment of COVID-19. *Ann. Glob. Health.* **2021**, *87*, 23.
15. Béché, E. Cameroonian responses to COVID-19 in the education sector: Exposing an inadequate education system. *Inter. Rev. Education.* **2020**:1–21.
16. Djoutsop, O.; Vanessa, Y.; Kanmounye, U.S. COVID-19 and Clinical Rotations in the Democratic Republic of Congo. *The International Journal of Medical Students.* **2020**, *9*.
17. Sasidharan, S.; Singh, V.; Babitha, M.; Dhillon, H. COVID19 - A report from the Democratic Republic of the Congo. *Dev. World Bioeth.* **2020**, *20*, 120–121.
18. Sasidharan, S.; Dhillon, H. Medical Training for Undergraduates in the Time of COVID-19: Education Cannot Wait in DR Congo. *Rev. Chil. Anest.* **2020**, *49*, 770–773.
19. Tsima, B.M.; Masupe, T.; Setlhare, V. Service-learning in response to the coronavirus disease 2019 pandemic: Emerging lessons from the Department of Family Medicine and Public Health at the University of Botswana. *Afr. J. Prim. Health Care Fam. Med.* **2020**, *12*, e1–e3.
20. Mlangeni, N. Building a resilient education system with digital solutions in Eswatini. 2020. Available at URL: https://www.sz.undp.org/content/eswatini/en/home/blog/building-a-resilient-education-system-in-eswatini.html (accessed 3 September 2021).
21. Chirwa J. Students relieved at planned reopening of universities. 2020. Available at URL: https://www.universityworldnews.com/post.php?story=20200616184759894 (accessed 4 September 2021).
22. Mukute, M.; Francis, B.; Burt, J.; de Souza, B. Education in Times of COVID-19: Looking for Silver Linings in Southern Africa's Educational Responses. *Southern African Journal of Environmental Education.* **2020**, *36*, 1–16.
23. Mavu, D.; Lates, J.; Hango, E.; Rennie, T.; Lusepani, M.; Kibuule, D.; Mubita. M. COVID-19 resilient pharmacy education: A grassroots work-integrated programme to strengthen the pharmaceutical workforce in Namibia. *Pharmacy Education.* **2020**, *20*, 205–11.
24. Kibuule, D.; Lates, J.; Ishola, A.; Hango, E.; Kalemeera, F.; Knott, M.; Mavu, D.; Singu, B.; Rennie, T.; Brinkmann, I.; et al. Migration to online pharmacy education in Namibia: Successes, challenges and competence implications. *Pharmacy Education.* **2020**, *20*, 174–82.
25. Kibuule, D.; Nambahu, L.; Sefah, I.A.; Kurdi, A.; Phuong, T.N.T.; Kwon, H-Y.; Godman, B. Activities in Namibia to Limit the Prevalence and Mortality from COVID-19 Including Community Pharmacy Activities and the Implications. *Sch. Acad. J. Pharm.* **2021**, *10*, 82–92.
26. Mavu, D.; Mubita, M.; Niaz, Q.; Lusepani, M.; Nowaseb, S.; Enkara, T.; Philomien, A.; Pick, R.; Kibuule, D. Emergency compounding of COVID-19 medicines: A readiness programme to up-skill pharmacy graduates in Namibia. *Pharmacy Education.* **2020**, *20*, 117–23.
27. Wangenge-Ouma, G.; Kupe, T. UNCERTAIN TIMES: Re-imagining universities for new, sustainable futures. August 2020. Available at URL: https://www.up.ac.za/media/shared/1/2020/September%202020/uncertain-times-paper_final_08092020-1.zp194357.pdf (accessed 4 September 2021).
28. David, W.H.; Michelle, G.; Gregory, D.B.; Werner, N.; Jansen van V, B. COVID-19 and the academe in South Africa: Not business as usual. *South African Journal of Science.* **2020**, *116*
29. Landa, N.; Zhou, S.; Marongwe N. Education in emergencies: Lessons from COVID-19 in South Africa. *Int. Rev. Educ.* **2021**, 1–17.
30. Bangalee, V.; Garza, O.; Oosthuizen, F.; Perumal-Pillay, V.; Rotundo, H. Teaching in the time of COVID-19: Shared perspectives from South Africa and the USA. *Afr. J Health Professions Educ.* **2021**, *13*.

31. Eksteen, M.J. EDITORIAL: Pharmacy workforce education challenges and responses to the COVID-19 pandemic in South Africa. *Pharmacy Education.* **2021**, *20*, 74–77.
32. Shereni, C. Covid-19 and challenges to education – Tell Zimbabwe. 2021. Available at URL: https://zifmstereo.co.zw/covid-19-and-challenges-to-education-tell-zimbabwe/3457/ (accessed 4 September 2021).
33. Afriyie, D.K.; Asare, G.A.; Amponsah, S.K.; Godman, B. COVID-19 pandemic in resource-poor countries: challenges, experiences and opportunities in Ghana. *Journal of infection in developing countries.* **2020**, *14*, 838–843.
34. Kawaguchi-Suzuki, M.; Nagai, N.; Akonoghrere, R.O.; Desborough, J.A. COVID-19 Pandemic Challenges and Lessons Learned by Pharmacy Educators Around the Globe. *American journal of pharmaceutical education* **2020**, *84*, ajpe8197.
35. Okoro, R.N.; Aluh, D.O.; Biambo, A.A.; Hedima, E.W.; Oluigbo, K.E. The impact of COVID-19 on pharmacy education: Perspectives of Nigerian undergraduate pharmacy students. *Pharmacy Education.* **2020**, *20*, 242–248.
36. Oladipo, A.T.; Fashola, O.T.; Agboola, E.I.; Adisa, O.O.; Oyekanmi, O.D.; Akinsete, A.M. Challenges with medical education in Nigeria in the COVID-19 era. *Pan Afr Med J.* **2020**, *37*, 223.
37. Gaur, U.; Majumder, M.A.A,; Sa, B.; Sarkar, S.; Williams, A.; Singh, K. Challenges and Opportunities of Preclinical Medical Education: COVID-19 Crisis and Beyond. *SN Compr. Clin. Med.* **2020**, 1–6.
38. Iwanaga, J.; Loukas, M.; Dumont, A.S.; Tubbs, R.S. A review of anatomy education during and after the COVID-19 pandemic: Revisiting traditional and modern methods to achieve future innovation. *Clinical anatomy.* **2021**, *34*, 108–114.
39. Onigbinde, O.A.; Chia, T.; Oyeniran, O.I.; Ajagbe, A.O. The place of cadaveric dissection in post-COVID-19 anatomy education. *Morphologie.* **2021**, *105*, 259–266.
40. Adeboyejo, A. COVID-19's Impact on Educational Inequality in Lagos State, Nigeria. 2021. Available at URL: https://covid19africawatch.org/pandemic-impacts-on-educational-inequality-in-lagos-state-nigeria/ (accessed 2 September 2021).
41. Solans, O.; Vidal-Alaball, J.; Roig Cabo, P.; Mora, N.; Coma, E.; Bonet Simó, J.M.; Hermosilla Pérez, E.; Saigí-Rubió, F.; Olmos Domínguez, C.; Piera-Jiménez, J.; et al. **C**haracteristics of Citizens and Their Use of Teleconsultations in Primary Care in the Catalan Public Health System Before and During the COVID-19 Pandemic: Retrospective Descriptive Cross-sectional Study. *J. Med. Internet Res.* **2021**, *23*, e28629.
42. Weber, A.M.; Dua, A.; Chang, K.; Jupalli, H.; Rizwan, F.; Chouthai, A.; Chen, C. An outpatient telehealth elective for displaced clinical learners during the COVID-19 pandemic. *BMC medical education.* **2021**, *21*, 174.
43. Olayiwola, J.N.; Udenyi, E.D.; Yusuf, G.; Magaña, C.; Patel, R.; Duck, B.; Sajanlal, S.; Potapov, A.; Kibuka C. Leveraging Electronic Consultations to Address Severe Subspecialty Care Access Gaps in Nigeria. *Journal of the National Medical Association.* **2020**, *112*, 97–102.
44. Kalungia, A.C.; Muungo, L.T.; Marshall, S.; Apampa, B.; May, C.; Munkombwe, D. Training of pharmacists in Zambia: Developments, curriculum structure and future perspectives. *Pharmacy Education.* **2019**, *19*, 69–78.
45. Mwila, K.; Kalolo, F.; Mudenda, S.; Hikaambo, C.N. Impact of COVID-19 on Academic Activities of Final Year Nursing Students in Zambia: Evidence from Zambia. Research Square. 2021. DOI: https://doi.org/10.21203/rs.3.rs-384363/v1 (accessed 3 September 2021).
46. Mudenda, S.; Mukosha, M.; Mwila, C.; Saleem, Z.; Kalungia, A.C.; Munkombwe, D. Impact of the coronavirus disease on the mental health and physical activity of pharmacy students at the University of Zambia: a cross-sectional study. *Int. J. Basic Clin. Pharmacol.* **2021**, *10*, 324–32.
47. Mwale, N.; Chita, J. Higher education and programme delivery in the context of COVID-19 and institutional closures: student responses to the adoption of e-Learning at a public university in Zambia. 2020, 9–33. Available at URL: http://alternation.ukzn.ac.za/Files/books/series-02/01-Mwale.pdf (accessed 3 September 2021).
48. Lates, J.; Kibuule, D. EDITORIAL: COVID-19: Rebooting pharmacy education in resource-limited settings. *Pharmacy Education.* **2021**, 67–70.
49. Chembe, C.; Kunda, D.; Simfukwe, M. Challenges and Benefits of Educational Roaming (eduroam) Service to ZAMREN Member Institutions. 2014. Availabable at URL: https://repository.ubuntunet.net/bitstream/handle/10.20374/144/chembec.pdf?sequence=1&isAllowed=y (accessed September 2021).
